# Screening the Medicines for Malaria Venture "Malaria Box" against the *Plasmodium falciparum* Aminopeptidases, M1, M17 and M18

**DOI:** 10.1371/journal.pone.0115859

**Published:** 2015-02-20

**Authors:** Alessandro Paiardini, Rebecca S. Bamert, Komagal Kannan-Sivaraman, Nyssa Drinkwater, Shailesh N. Mistry, Peter J. Scammells, Sheena McGowan

**Affiliations:** 1 Dipartmento di Scienze biochimiche "A. Rossi Fanelli", Sapienza Università di Roma, Roma, Italy; 2 Department of Biochemistry and Molecular Biology, Monash University, Clayton Campus, Melbourne, Victoria, Australia; 3 Medicinal Chemistry, Monash Institute of Pharmaceutical Sciences, Monash University, Parkville Campus, Melbourne, Victoria, Australia; Tulane University, UNITED STATES

## Abstract

Malaria is a parasitic disease that remains a global health burden. The ability of the parasite to rapidly develop resistance to therapeutics drives an urgent need for the delivery of new drugs. The Medicines for Malaria Venture have compounds known for their antimalarial activity, but not necessarily the molecular targets. In this study, we assess the ability of the “MMV 400” compounds to inhibit the activity of three metalloaminopeptidases from *Plasmodium falciparum*, *Pf*A-M1, *Pf*A-M17 and *Pf*M18 AAP. We have developed a multiplex assay system to allow rapid primary screening of compounds against all three metalloaminopeptidases, followed by detailed analysis of promising compounds. Our results show that there were no *Pf*M18AAP inhibitors, whereas two moderate inhibitors of the neutral aminopeptidases *Pf*A-M1 and *Pf*A-M17 were identified. Further investigation through structure-activity relationship studies and molecular docking suggest that these compounds are competitive inhibitors with novel binding mechanisms, acting through either non-classical zinc coordination or independently of zinc binding altogether. Although it is unlikely that inhibition of *Pf*A-M1 and/or *Pf*A-M17 is the primary mechanism responsible for the antiplasmodial activity reported for these compounds, their detailed characterization, as presented in this work, pave the way for their further optimization as a novel class of dual *Pf*A-M1/*Pf*A-M17 inhibitors utilising non-classical zinc binding groups.

## Introduction

Malaria is a tropical disease that is caused by infection of the protozoan parasites of the genus *Plasmodium*. In 2013, malaria was responsible for over 600,000 deaths with the majority of mortality seen in children under the age of five [1]. The discovery of new antiplasmodial agents is necessary to overcome the spread of malaria parasites that have become resistant to currently available drugs [2]. Global efforts continue to develop efficacious medicines that are affordable, accessible and appropriate for use in areas endemic with malaria. To catalyse drug discovery and research, the non-profit foundation Medicines for Malaria Venture (MMV) and SCYNEXIS have provided a compound library (MMV400) for the research community [3]. These compounds are known for their antimalarial activity but their molecular targets and mode of action are not necessarily known. All of the compounds have confirmed activity against the asexual blood-stage of *Plasmodium falciparum (Pf)* and are commercially available. The MMV400 'Malaria Box' contains 200 drug-like compounds (for oral drug discovery and development) and 200 probe-like compounds for use as biological tools in malaria research. The compounds have been tested for cytotoxicity and are within levels considered acceptable for an initial drug discovery programme [3].

The release of the MMV400 box to the wider community was to encourage the identification of novel molecular targets for further target validation and compound optimisation. We have used the MMV400 Box to screen against three of the malarial metallo-aminopeptidases: *Pf*A-M1 (Clan MA), *Pf*A-M17 (Clan MF) and *Pf*M18AAP (Clan MF), to determine if any of the MMV400 compounds were active against these enzymes. The three metalloaminopeptidases, referred to collectively in this study as the *Pf*MAPs, are emerging as targets for novel antimalarials [4–6]. These enzymes catalyse the cleavage of N-terminal amino acids from proteins and peptides. Given the restricted specificities of each of these enzymes for different N-terminal amino acids, it is thought that they act in concert to facilitate protein turnover [4–6]. All three PfMAPs are expressed through all stages of the intra-erythrocytic life-cycle of the parasite and can be located in the cytosol of the parasite [6–8]. *Pf*A-M1 also functions within the digestive vacuole [9] and was recently found in the nucleus [10]. The role of the nuclear form of the enzyme remains unknown. The monomeric *Pf*A-M1 is an essential hemoglobinase involved in the final steps of hemoglobin digestion, a process that provides essential free amino acids to the parasite for its own protein anabolism [9]. The role of *Pf*A-M1 is therefore provision of essential nutrients to the parasite. There is much interest in the development of inhibitors to *Pf*A-M1 that could act as lead compounds for drug development [11–14]. The hexameric *Pf*A-M17 and dodecameric *Pf*M18AAP have also been implicated in hemoglobin digestion; however, recent studies have suggest both enzymes play additional roles in the parasite, including important housekeeping functions and in conjunction with the parasite proteosome [8,9,15].

We developed a high-throughput approach for a primary screen, where we could assay three enzymes against a single compound in a single well, allowing us to rapidly triage the 400 compounds. We followed this primary screen with a secondary screen to identify and confirm compounds that reduced the activity of the *Pf*MAPs. Individual activity of the enzymes was assessed and key identified compounds were docked into the active site of the enzyme to gain additional structural insights into the inhibition process of the *Pf*MAPs by the identified inhibitors. Here, we present the results of the biochemical screen and the related structure-activity relationship data.

## Materials and Methods

### Recombinant protein production

The production of *Pf*A-M1 and *Pf*A-M17 in *Escherichia coli* and purification using a two-step purification process of Ni-NTA-agarose column followed by size exclusion chromatography on a Superdex 200 16/60 using a AKTAxpress high throughput chromatography system (http://proteinexpress.med.monash.eud.au/index.htm) was as described [5,6] The production of *Pf*M18AAP, including dissociation from GroEL, was also as described [15].

### Multiplex enyzme assays

A multiplex enzyme assay was developed to complete the primary screen of 400 compounds that were provided to us by MMV and SCYNEXIS. Combined *Pf*MAP (15 nM *Pf*A-M1; 80 nM *Pf*A-M17 and 125 nM *Pf*M18AAP) aminopeptidase activity in the presence of a single MMV compound (100 μM solubilized in 100% dimethyl sulfoxide, DMSO, final DMSO concentration in assay = 10%) was determined by simultaneous measurement of the release of the fluorogenic leaving group, -NHMec, from L-Leucine-7-amido-4-methylcoumarin-HCl (Leu-Mec) (Sigma) as well as the hydrolysis of *p*-nitroanilide from the chromogenic substrate, L-Glutamic acid *p*-nitroanilide (Glu-pNA) (Sigma). The fluorogenic substrate records the combined activity of *Pf*A-M1 and *Pf*A-M17 while the *p*-nitroanilide substrate is specific for *Pf*M18AAP. Reactions were carried out in 96-well microtitre plates (200 μL total volume) in 50 mM Tris-HCl pH 8.0, 2 mM CoCl_2_ at 37°C. Following a 10 min incubation (multi-enzyme and single compound) at 37°C, reactions were initiated by addition of both fluorogenic and chromagenic substrates. Progress curves were monitored using a FLUOstar Omega multimode reader (BMG LabTech) for 1.5 h at 37°C. As activity references, individual enzyme and multiple enzyme 'no compound' controls and 'positive inhibitor' controls (with 10% DMSO) were included in each 96 well plate assay. The positive control inhibitors selected were Bestatin, a Phe-Leu dipeptide analog known to potently inhibit *Pf*A-M1 and *Pf*A-M17 [5,6] and 2-benzyl-4,6-dinitrobenzo[*d*]isothiazol-3(2*H*)-one which we have shown to inhibit *Pf*M18AAP.

### Secondary screening of MMV compounds against individual enzymes

Aminopeptidase activity of each individual enzyme in the presence of a single MMV compound (100 μM) was determined using Leu-Mec for both *Pf*A-M1 (15 nM) and *Pf*A-M17 (80 nM) or Glu-pNA for *Pf*M18AAP (125 nM). Reactions were carried out in 96-well microtitre plates (200 μl total volume) in 50 mM Tris-HCl pH 8.0, 2 mM CoCl_2_ at 37°C. Following a 10 min incubation (enzyme and compound) at 37°C, reactions were initiated by addition of enzyme specific substrate. Progress curves were monitored using a FLUOstar Omega multimode reader (BMG LabTech) for 1.5 h at 37°C. As activity references, individual enzyme 'no compound' controls and 'positive inhibitor' controls were included in each 96 well plate assay. We applied a cut-off of < 10% activity in the presence of 100 μM compound for selection to the next phase. Compounds that were selected to proceed were then used in further assays to obtain n = 3.

### Determination of *K*_i_ values for purchased compounds

MMV020750 was purchased from InterBioScreen (catalog number STOCK2S-51608) and MMV666023 was purchased from ChemBridge (catalog number 5566248). The MMV020750 derivatives **1**, **2** and **3** were purchased from Ambinter (catalog numbers AMB425981, AMB2311364 and AMB8506251 respectively). 4-(4,5-Diphenyl-1*H*-imidazol-2-yl)benzoic acid (**4**) was purchased from TCI Chemicals, while 4-(4,5-diphenyl-1*H*-imidazol-2-yl)-*N*-hydroxybenzamide (**5**) was prepared from **4** as described (S1 Methods). Aminopeptidase activity of both *Pf*A-M1 and *Pf*A-M17 enzymes was determined by measuring the release of the fluorogenic leaving group, NHMec, from the fluorogenic peptide L-Leucine-7-amido-4-methylcoumarin hydrochloride (H-Leu-NHMec) (Sigma L2145). The assay protocol devised by Stack CM, *et al*, (*J*. *Biol Chem*, 2007) was modified and used, with the reactions were carried out at 37°C and monitored using a spectrofluorometer (BMG FLUOstar) with excitation at 355 nm and emission at 460 nm. Each enzyme was pre-incubated in 50 mM Tris pH 8.0, 2 mM CoCl_2_; 5% DMSO with each inhibitor for 10 min prior to the addition of substrate. Inhibitor concentration was assayed between 0 and 500 μM final for each compound, and repeated over a narrowed range if necessary. The fluorescence signal was monitored until a final steady state velocity, V, was obtained. The *K*_i_ values were then evaluated using Dixon plots of 1/V versus inhibitor concentration while the substrate concentration was maintained lower than that of the K_*m*_ of the enzyme.

Analysis of **4** via these methods proved problematic due to the presence of an intrinsic fluorescence in the compound. This was a particular problem with the *Pf*A-M17 enzyme assays where we noted that at higher concentrations of **4**, a decrease in fluorescence was observed. We hypothesise that this is a result of *Pf*A-M17 binding to the compound and quenching the intrinsic fluorescence. We attempted to overcome the problem by the use of L-Leucine *p*-nitroanilide (Sigma); however, the activity of *Pf*A-M17 (K_*m*_/kcat) is significantly reduced with this substrate. We therefore chose to analyse a reduced concentration range for **4**.

### Molecular Docking of selected hits

Molecular docking was carried out by means of Molegro Virtual Docker (MVD) software (CLCbio). Ligands were built and energy minimized by using the PRODRG server [16]. Flexible torsions were automatically detected by MVD, and manually checked for consistency. The three-dimensional structures of *Pf*A-M1 (PDB Code: 3EBH; [6]) and *Pf*A-M17 (PDB Code: 3KR4; [5]) in complex with Bestatin were prepared by automatically assigning bond orders and hybridization, and adding explicit hydrogens, charges and Tripos atom types. Crystallographic water molecules and Bestatin were ignored and excluded from docking computations. Missing heavy atoms were fixed by modeling them, using Modeler v.9.8 [17] and PyMod [18]. A search space of 15Å radius, centered on the ligand Bestatin, was used for docking. Grid-based MolDock score [19] with a grid resolution of 0.30 Å was used as scoring function for docking. MolDock SE was used as docking algorithm. For each ligand, ten runs were defined. Similar poses (RMSD < 1.2 Å) were clustered, and the best scoring one was taken as representative. Other docking parameters were fixed at their default values. After docking, energy optimization of hydrogen bonds was performed. The “Affinity” score of MVD was used to re-score the obtained poses and estimate free energy of binding.

## Results

### Primary Screen of the MMV400 Box

To reduce the materials required to screen all 400 compounds against three different enzymes, we developed a multiplex assay system that allowed us to screen a single compound against all three *Pf*MAPs in one well of a 96 well tray. During our development of this assay we determined that a single assay buffer was compatible with all three *Pf*MAPs and that the individual activities are independent and unaffected by the presence of the other *Pf*MAPs. We further tested the combination of substrates required to allow for the individual specificities of each enzyme and designed a combination of laboratory inhibitors to be used as a "positive for inhibition" controls. A fluorogenic substrate was used to monitor the combined activity of *Pf*A-M1 and *Pf*A-M17 and a chromogenic substrate was used for *Pf*M18AAP. The combination of substrates meant that the primary screen would immediately identify *Pf*M18AAP inhibitors; however, any inhibitors of *Pf*A-M1 or –M17 would require a deconvolution step to identify which of the two enzymes the inhibitory compound was targeting.

**Fig 1:**
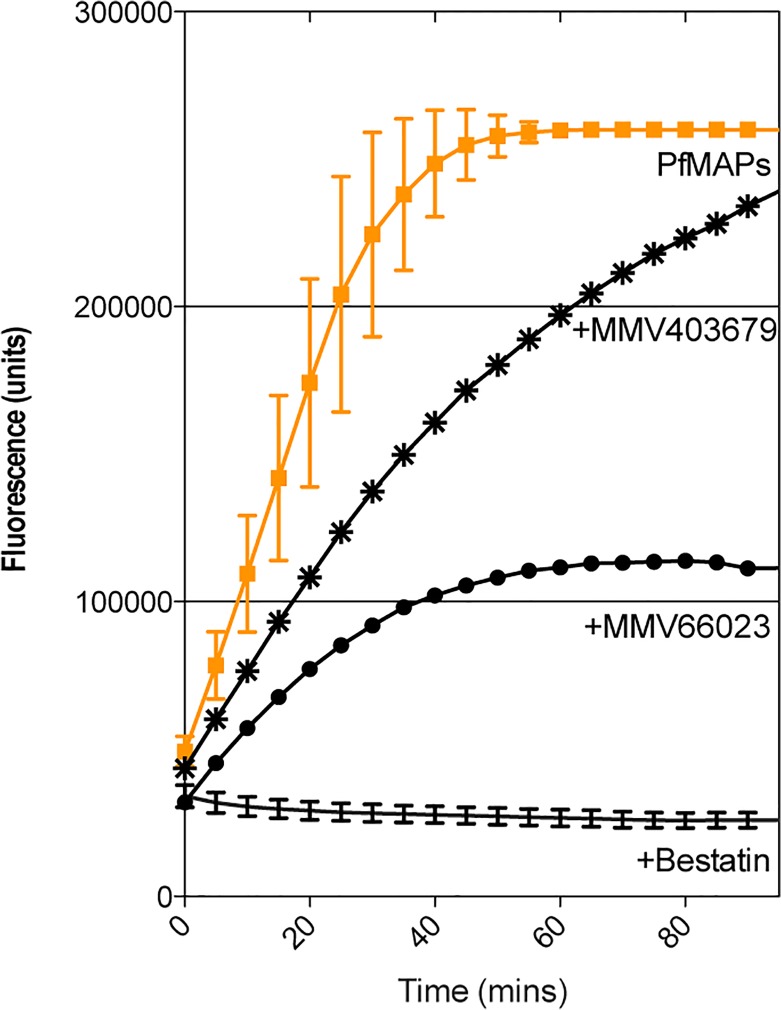
Representative progress curves from multiplex assay screening *Pf*A-M1 and –M17 against the MMV400 library. The multiplex assay screened one compound (100 μM) against three enzymes. The activity of *Pf*A-M1 and—M17 were measured by change in fluorescence units over time. No inhibitor controls (n = 8) for *Pf*MAP activity were included in each plate screened (shown in orange). A representative of the ‘drug-like’ compounds (MMV403679) is indicated by asterisk (*). A ‘probe-like’ compound (MMV666023) by solid dots (•). The inhibitor control (Bestatin) is shown as a solid black line.

The MMV400 Box is stored and shipped in 96 well plates with 80 compounds per tray. This leaves 16 wells to ensure that each plate has control assays included. To get accurate and robust data from our primary screen, we included each enzyme separately to ensure that each individual enzyme was active, a "positive for inhibition" control to observe 100% inhibition and 8 *Pf*MAP 'no compound' controls to provide reliable data with which to assign "100% activity". Our analysis of the primary screen involved visual inspection of the assay progress curves. If a progress curve was within the error range of the *Pf*MAP activity curve (Fig. 1) then that compound was deemed non-inhibitory, as it did not affect the activity of the enzymes. This allowed quick identification of compounds that showed an apparent reduced enzyme velocity (progress curves that lay outside of error margins). A representative assay is shown in Fig. 1 and a complete summary of results can be found in S1 Fig.

From the primary screen, 25 compounds reduced the fluorescence released in the assay, indicating that they were interfering with either *Pf*A-M1 and/or *Pf*A-M17 activity, however no change in absorbance, indicative of *Pf*-M18 inhibition, was observed (S1 Fig.). There are therefore no *Pf*M18AAP inhibitors present in the MMV400 Box. Of the 25 hit compounds against *Pf*A-M1 and/or *Pf*A-M17, three are ‘drug-like’, and the remaining 22 ‘probe-like’. Although the reduction in activity for all 25 compounds was modest, MMV666023 was identified as the compound that exhibited the most potent inhibitory effect (Fig. 1).

### *Pf*A-M1 and *Pf*A-M17 inhibitors identified in MMV400 Box

**Fig 2:**
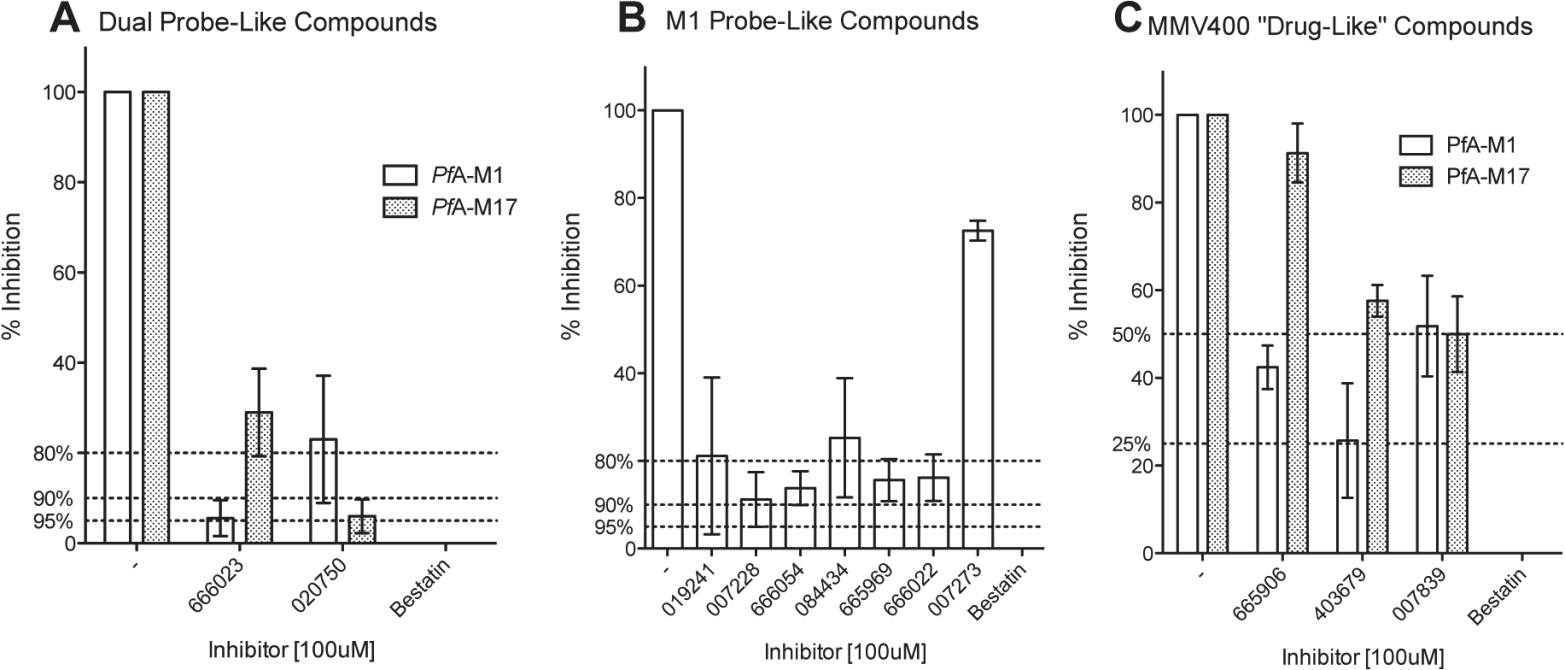
Inhibitory activity of selected MMV compounds against *Pf*A-M1 and *Pf*A-M17. Enzyme activity in the presence of 100 μM compound (MMV# shown) was compared to activity of the enzyme in the absence of any inhibitor (-). An inhibitor control using Bestatin was included and no neutral aminopeptidase activity is detectable in the presence of 100 μM Bestatin. A dashed line is shown to indicate the when the activity of either enzyme was reduced by 95, 90 and 80% or more. The activity of *Pf*A-M1 is shown as clear boxes and *Pf*A-M17 as hashed boxes. (**A**) Compounds that appeared to show dual activity against both enzymes. (**B**) Compounds that showed activity against *Pf*A-M1 and (**C**) ‘drug-like’ compounds that showed limited activity against either enzyme.

We subsequently screened the 25 compounds (at 100 μM) against *Pf*A-M1 and *Pf*A-M17 in separate assays to determine which of the enzymes each compound was targeting. From this preliminary single dose secondary screen, we eliminated MMV665840 as a false positive from the primary screen. Given the reduction in activity was modest for all compounds at 100 μM, we looked for the most potent of the 24 remaining compounds (S2 Fig., n = 1). Applying a cut-off of < 10% activity in the presence of 100 μM compound (S2 Fig.), we selected nine compounds, two putative dual inhibitors (affecting *Pf*A-M1 and *Pf*A-M17) and seven putative *Pf*A-M1 inhibitors. We also chose to retain our three 'drug-like' hits for the next round. We then proceeded to assay each of the selected compounds against *Pf*A-M1 and *Pf*A-M17 (Fig. 2, n = 3) to obtain reliable data on their inhibitory properties.

The putative dual inhibitors were the most potent inhibitors identified in the screen for both enzymes (Fig. 2A). MMV666023 was able to reduce the activity of *Pf*A-M1 by 95% and *Pf*A-M17 by 70%. MMV020750 had the opposite specificity, and was able to reduce *Pf*A-M17 by 95% whilst reducing *Pf*A-M1 activity to only 75% of total enzyme activity (Fig. 2A). The results for MMV020750 show the importance of robust investigation as the selectivity of this compound (*Pf*A-M1 vs *Pf*A-M17) was reversed when the biological replicates were completed, in comparison to the single dose, single assay data (S2 Fig.). The compounds that reduced *Pf*A-M1 activity to < 10% in the secondary screen (S2 Fig.) were not as inhibitory as MMV666023. MMV007273 was deemed to be a false positive, retaining nearly 75% activity in the presence of the compound (Fig. 2B). The most potent in this group of compounds were MMV007228 and MMV666054 that reduced *Pf*A-M1 activity by 89% and 87%, respectively (Fig. 2B). As predicted in the primary and secondary screens, the 'drug-like' compounds were less effective in targeting either *Pf*A-M1 or *Pf*A-M17 (Fig. 2C). MMV403679 was able to reduce the activity of *Pf*A-M1 by 75% (Fig. 2C).

**Fig 3:**
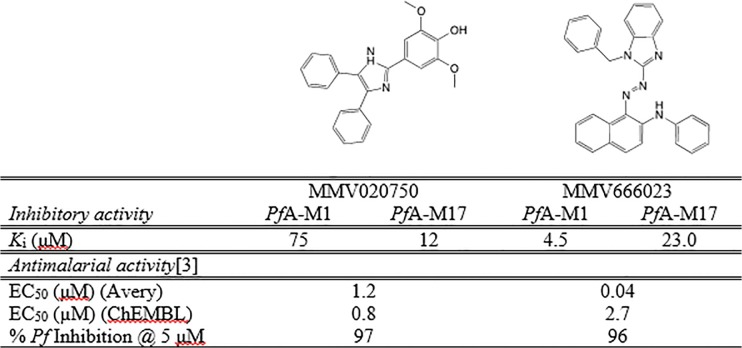
MMV020750 & MMV666023 inhibitor constants.

The most potent inhibitors of *Pf*A-M1 and *Pf*A-M17 were MMV666023 and, MMV020750, respectively. We undertook a further analysis of these two inhibitors against each protein. To complete further assays we needed to purchase the two compounds from commercial suppliers. To enable us to determine the mode of inhibition, we measured *K*_i_ values for both inhibitors versus each enzyme (Fig. 3, S3 Fig.). We experimentally determined these values and showed that the inhibitors were acting on *both* enzymes, as competitive inhibitors, with micromolar inhibitory activity (S3 Fig.). Although the identified compounds inhibit both *Pf*A-M1 and *Pf*A-M17, our results indicate that MMV020750 and MMV666023 preferentially inhibit one of the two enzymes. MMV020750 was a better inhibitor of *Pf*A-M17 (6-fold selectivity over M1) whilst MMV666023 was a moderate inhibitor of *Pf*A-M1 (5-fold selectivity over M17) (Fig. 3).

### Docking of the inhibitors to the active site of *Pf*A-M1 and *Pf*A-M17

*Pf*A-M1 and *Pf*A-M17 both contain essential zinc ions in their respective active sites. To date, chelation of the zinc ions through a zinc-binding group (ZBG) is the most common mode of action for competitive inhibitors targeting these enzymes [20,21]. However, MMV666023 and MMV020750 show no classical ZBG. To elucidate how these compounds inhibit *Pf*A-M1 and *Pf*A-M17 we chose to investigate the mechanism of binding to both enzymes through molecular docking calculations.

**Fig 4:**
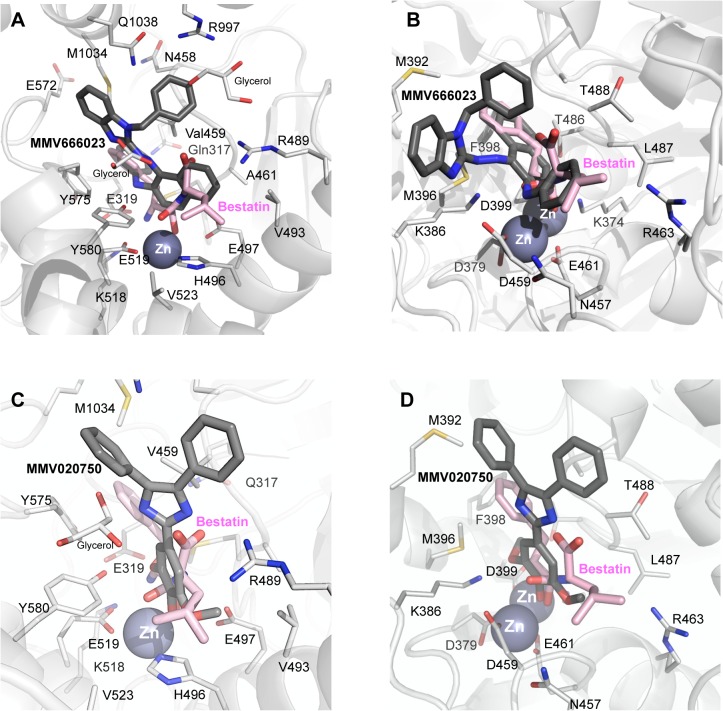
Diagram of MMV666023 (top) and MMV020750 (botttom) binding to active sites of *Pf*A-M1 (A, C) and *Pf*A-M17 (B, D). Carbon atoms of *Pf*A-M1/*Pf*A-M17 residues and the inhibitor are colored in light and dark gray, respectively. Zinc ions are shown as spheres. Bestatin is colored in pink.

The highly hydrophobic MMV666023 is predicted to occupy similar space in the active site to that observed by the well-characterised inhibitor, Bestatin, in *Pf*A-M1 crystal structures [6,9,14] (Fig. 4A). The naphthalene and benzimidazole moieties span from the S1’ to the S3’ sub-sites, respectively, without coordinating the zinc ion. Two additional clefts, in which two glycerol moieties are present in the crystal structure of *Pf*A-M1 in complex with Bestatin [6] are occupied by the N4- and N5- linked aromatic rings. Hydrophobic interactions are observed between MMV666023 and residues Val459, Tyr575, Tyr580, Met1034, Gln1038 and Arg489. MMV666023 binding is further stabilized by a hydrogen-bond interaction with Tyr580. The binding mode of MMV666023 to the active site of *Pf*A-M17 resembles, again, the observed mode for Bestatin [5], with the hydrophobic rings interacting with residues Met392, Met396, Phe398 and Leu487, and an additional hydrogen bond with the main-chain of residue Asp459 (Fig. 4B). As for *Pf*A-M1, no coordination of the zinc ion is predicted to take place. The predicted binding free energies of MMV666023 with *Pf*A-M1 and *Pf*A-M17 averaged -4.91 kcal/mol (-5.22 kcal/mol and -4.61 kcal/mol, respectively), corresponding to a predicted low-medium micromolar constant of inhibition, in agreement with the observed experimental data.

The hydroxyl group of syringol (1,3-dimethyl pyrogallate) moiety of MMV020750 is docked at coordinating distance (~ 2.1 Å) from the zinc ion of *Pf*A-M1 (Fig. 4C). A hydrogen bond is formed between Tyr580 and the O1 atom of the pyrogallate moiety. Another stabilizing interaction is represented by the hydrogen bond between the Gly460 main chain and N1 atom of the 2,4,5-triphenyl-1*H*-imidazole moiety. Likewise, the docked complex between MMV020750 and *Pf*A-M17 (Fig. 4D) suggests that the hydroxyl group of syringol is placed at coordinating distance (~2.4 Å) with the two zinc atoms (Fig. 4D). A hydrogen bond is formed between Lys386 and the O1 atom of the pyrogallate moiety. Again, the predicted binding free energies of MMV020750 with *Pf*A-M1 and *Pf*A-M17 averaged -4.09 kcal/mol (-4.42 kcal/mol and -3.76 kcal/mol, respectively), corresponding to a predicted medium low-micromolar constant of inhibition.

### Structure-Activity-Relationships of MMV020750

**Fig 5:**
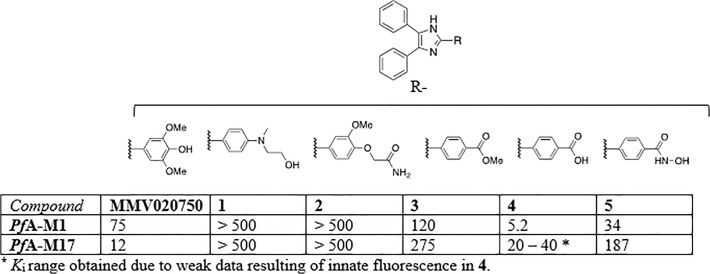
Inhibition of *Pf*A-M1 and *Pf*A-M17 by derivatives of MMV020750.

The hydrophobic MMV666023 was considered sub-optimal for further investigation and development due to its highly lipophilic nature (AlogP = 8.5, [3]) and the presence of the azo group, that is known to produce toxic metabolites. However, given the predicted novel binding mode of MMV020750, which showed coordination of the zinc ion/s by non-classical ZBGs, we were interested in investigating the structure-activity relationships (SAR) of the compound further. To attempt to uncover SAR surrounding MMV020750, we choose to use the 2,4,5-triphenyl-1*H*-imidazole as a core scaffold and investigated the role of the 2-phenyl substituent (Fig. 5). It was rationalised that the *ortho*-methoxyphenol moiety present on MMV020750 may be coordinating to a zinc ion in the active site of the enzymes, so a range of potential zinc-binding groups were assessed in this position. Accordingly, the substituents that where chosen all possessed donor atoms and also included the well-known hydroxamic acid zinc-binding functionality (see Fig. 5). These substituents also had the potential of establishing other interactions with the active site (e.g. hydrogen bonding interactions) and revealing additional SAR.

The *K*_i_ values of these compounds were determined and compared to that of the parent compound, MMV020750 (Fig. 5, S4 Fig., and S5 Fig.). The analogues with a larger substituent in the *para*-position of the 2-phenyl group (namely compounds **1** and **2**), exhibited no inhibitory effect on either *Pf*A-M1 or *Pf*A-M17 up to 500 μM, suggesting that an unfavourable steric interaction may have been introduced. The remaining compounds possessed a carboxylic acid or derivative in the *para*-position of the 2-phenyl group. Of these, the ester **3** and the hydroxamic acid **5** showed similar behaviour to that of the parent compound with inhibitory activity in the micromolar range against *Pf*A-M1, but a > 10-fold loss in inhibitory activity against *Pf*A-M17. The only exception to this was the activity of the carboxylic acid **4**, where we noted a > 10-fold increase in potency against *Pf*A-M1. Analysis of inhibitory activity of **4** against *Pf*A-M17 was affected by technical problems and our results indicate a *K*_i_ in the micromolar range.

MMV020750 derivatives were docked by using the obtained pose of MMV020750 as a structural template (Fig. 4 C and D). Compounds **1** and **2** (which have longer substituents at the *para-* position of the 2-phenyl ring) were unable to coordinate the zinc ion(s) of either enzyme (S4 Fig. and S5 Fig.). In *Pf*A-M1, steric clashes were observed with residues surrounding the active site pocket (His496, Glu497, His500 and Glu519). Similarly, with *Pf*A-M17, steric clashes were observed with residues Asp379, Asp399, Asp459 and Glu461 that act to coordinate the two zinc ions in the active site (S4 Fig.). This observation accounts for the inability of **1** or **2** to inhibit either enzyme.

Conversely, the ZBG of compounds **3**–**5** were all placed within coordinating distance from the zinc ions of *Pf*A-M1 and *Pf*A-M17, while the 2,4,5-triphenyl-1*H*-imidazole scaffold retained a binding mode similar to MMV020750. Interestingly, both oxygen atoms of the carboxylic acid group of compound **4** were placed at coordinating distance from the Zn^2+^ ion of *Pf*A-M1, resulting in a shift from a monodentate, slightly distorted tetrahedral geometry (e.g., as observed for parent compound MMV020750), to a bidentate trigonal bipyramidal one (S5 Fig.). The stronger bidentate interaction between the active site Zn^2+^ ion and the carboxylate group could therefore be responsible for the observed > 10-fold increase in potency of **4** against *Pf*A-M1, compared to MMV020750. However, in contrast to MMV020750, a stabilizing hydrogen-bond interaction with Tyr580 is missing in compound **4**.

Compound **4** bound *Pf*A-M17 in a similar manner to the parent MMV020750, but each oxygen atom of the carboxylic acid group of **4** was placed within coordinating distance from its nearest Zn^2+^ ion (O1-Zn1 ~ 1.7 Å and O2-Zn2 ~ 2.1 Å), giving a monodentate binding to each catalytic zinc. Interestingly, coordination geometry varies between the two Zn^2+^ atoms, with one being coordinated with a tetrahedral geometry, and the other in a trigonal bipyramidal penta-coordination. The negatively charged carboxylic group of **4** may also be stabilized by an ion-pair interaction with the active site Lys374 (O1-Nε ~ 4.9 Å); although a hydrogen bond between Lys386 and the pyrogallate moiety is lost.

## Discussion

Recent years have seen an ‘open collaboration’ in the search for new drugs for neglected tropical diseases. Extensive libraries of compounds with varying biological activity have been made publically available, providing a valuable resource for research from a variety of disciplines. One such initiative is the ‘Malaria Box’ from MMV. The box comprises 200 drug-like compounds and 200 probe-like compounds with antimalarial activity. The drug-like compounds have physicochemical properties that are compliant with Lipinski’s ‘rule of 5’ (RO5) [22], with known toxicophores removed. Though their oral bioavailability remains untested, these compounds are considered good starting points for lead optimisation. The 200 probe-like compounds were included to use as tools for probing biological mechanisms of action against a particular target. These compounds are not necessarily RO5 compliant, but represent a broad cross-section of structural diversity. The MMV400 library was independently tested against the asexual blood stage of 3D7 *Pf*. The selected compounds were further tested for cytotoxicity and all show at least a 10-fold separation between the antimalarial and cytotoxicity results.

Here we present the results of our screen of the MMV400 library against the malarial aminopeptidases *Pf*A-M1, *Pf*A-M17 and *Pf*M18AAP. We developed a multiplex assay to triage the library against *Pf*MAPs activity, and demonstrated that it to be a highly sensitive and robust screening platform; it was able to rapidly identify even weak inhibitors with a low rate of false positives. From this assay, 3 of the ‘drug-like’ and 21 of the ‘probe-like’ compounds met our criteria for hit molecules targeting *Pf*A-M1 and/or *Pf*A-M1, and were selected for further investigation. In contrast, no *Pf*M18AAP inhibitory activity was observed for any of the compounds tested, which is likely due to the highly restricted specificity of *Pf*M18AAP [15]. A secondary screen was used to investigate the 24 hit compounds, and determined that at 100 μM, seven compounds inhibit the activity of either *Pf*A-M1 or *Pf*A-M17 by more than 90%, and two showed greater than 95% inhibition. The two most potent compounds, MMV666023 and MMV020750, were determined to be micromolar, competitive inhibitors of both *Pf*A-M1 and *Pf*A-M17.

*Pf*A-M1 and *Pf*A-M17 both contain essential zinc ions in their active sites, chelation of which has been the most common method of inhibition employed to target these enzymes. Given the absence of any classical zinc binding groups in either MMV666023 or MMV020750, we elected to structurally investigate their mechanism of binding to *Pf*A-M1 and *Pf*A-M17. Our attempts to co-crystallise the compounds proved unsuccessful so we performed molecular docking to investigate potential interactions the compounds might make with the active site of each enzyme. This approach was challenging due to the hydrophobic nature of the compounds and lack of a classical zinc-binding moiety on either compound. Consistent with this, we were unable to find any poses of MMV666023 in the docking results with atoms coordinating the zinc ion(s). If we tried to force zinc docking by imposing a zinc binder, highly unfavourable interactions within the active sites were introduced. We admit that this might be a bias of the docking procedure, which cannot consider major structural conformational changes of the active site, a property common to proteases.

Conversely, we have shown that the hydroxyl group of syringol moiety of MMV020750 can be placed at coordinating distance from the zinc ion(s) of *Pf*A-M1/ *Pf*A-M17. In agreement with these data, the replacement of the *para*-hydroxyl moiety of syringol with bulkier substituents results in a complete loss of affinity for *Pf*A-M1/*Pf*A-M17, while the replacement with classical zinc-binding groups results in a comparable affinity. We conclude that MMV020750 and its derivatives **3**–**5**, are acting as putative zinc-binding inhibitors.

The most potent current series’ of *Pf*A-M1 and *Pf*A-M17 inhibitors to date bind the enzyme active sites predominantly through coordination of the essential zinc ion/s. However, there are a number of drawbacks that are commonly associated with this mechanism, particularly low specificity for the target enzyme, and poor pharmacokinetics and oral bioavailability. Issues with compound specificity can be overcome by rational selection of the ZBG; optimisation of the ZBG itself has even been reported to impart improved selectivity on an inhibitor series [23]. However, poor pharmacokinetics and oral bioavailability are more difficult to overcome. Previous *Pf*A-M1 and *Pf*A-M17 inhibitor series developed within our group have utilised phosphonic acids as the ZBG [12], which could potentially be detrimental for compound membrane permeability. The discovery of novel compounds that bind *Pf*A-M1 and *Pf*A-M17 through a different ZBG, therefore has implications for the development of alternative metalloaminopeptidase inhibitors in addition to the MMV020750 series. Further, the finding that MMV666023 is unlikely to inhibit *Pf*A-M1 and *Pf*A-M17 by coordination of the zinc ion/s suggests that compounds that function completely independently of zinc binding could be identified.

## Conclusions

In summary, we have developed a sensitive and robust multiplex assay system to allow rapid primary screening of compounds against three emerging targets for novel antimalarials, *Pf*A-M1, *Pf*A-M17 and *Pf*M18. The MMV400 'Malaria Box' consisting of 200 drug-like compounds and 200 probe-like compounds, was then screened to determine if any of the MMV400 compounds were active against these three enzymes. A total of 9 small molecules were identified as micromolar inhibitors of *Pf*A-M1 and *Pf*A-M17. The mode of action of the most potent compounds, acting as dual inhibitors, was investigated. While MMV020750 coordinates the essential zinc ion/s of *Pf*A-M1 and *Pf*A-M17 through a non-classical ZBG, MMV666023 binds solely through interactions with active site residues, apparently acting completely independently of any ZBG. In conclusion, derivatives of MMV020750 may represent a starting point for finding potent molecules able to act on a fundamental pathway of *Plasmodium* life cycle.

## Supporting Information

S1 Fig Primary screen of MMV400 using a multiplex aminopeptidase assay. Enzyme activity that was reduced in comparison to control wells are indicated by “R” and compounds that had no effect on activity are shown as “UC” for unchanged. Compounds highlighted in grey are “drug-like” and the remainder are “probe-like”.
(PDF)

S2 Fig Secondary screen of *Pf*A-M1 and *Pf*A-M17 against 24 preliminary screen ‘hits’. Enzyme activity in the presence of 100 μM compound (MMV# shown) was compared to activity of the enzyme in the absence of any inhibitor (-). An inhibitor control using Bestatin was included and no neutral aminopeptidase activity is detectable in the presence of 100 μM Bestatin. A dashed line is shown to indicate the when the activity of either enzyme was reduced by 90% or more.
(PDF)

S3 Fig Inhibitory properties of MMV666023 and MMV020750. **(A)** Enzyme activity in the presence of increasing inhibitor concentration. Numbers shown on curves are inhibitor concentration in μM. (**B**) Dixon plot for calculation of *K*i where S1 and S2 are two different substrate concentrations (<< *K*M of enzyme).
(PDF)

S4 Fig Dixon plots and docking for MMV020750 derivatives with *Pf*A-M1. Dixon plots of *K*i data shown in Fig. 5 (*K*i defined as point of intersection and indicated by dotted line). Two different substrate concentrations are shown (solid circles and squares). Outliers not included in linear regression are shown as hollow squares or circles. 3D molecular docking diagrams shown with carbon atoms of *Pf*A-M17 residues and the inhibitor are colored in light and dark gray, respectively. Zinc ions are shown as spheres. Corresponding 2D molecular docking representations shown on right hand panel.
(PDF)

S5 Fig Dixon plots and docking for MMV020750 derivatives with *Pf*A-M17. Dixon plots of *K*i data shown in Fig. 5 (*K*i defined as point of intersection and indicated by dotted line except in **4** where a grey shaded area defines *K*i range). Two different substrate concentrations are shown (solid circles and squares). Outliers not included in linear regression are shown as hollow squares or circles. 3D molecular docking diagrams shown with carbon atoms of *Pf*A-M17 residues and the inhibitor are colored in light and dark gray, respectively. Zinc ions are shown as spheres. Corresponding 2D molecular docking representations shown on right hand panel.
(PDF)

S1 Methods Chemistry Experimental. (PDF)

## References

[1] World Health Organisation (2013) World Malaria Report 2013. WHO Library Cataloguing-in-Publication Data. Available: http://www.who.int/malaria.

[2] Ariey F, Witkowski B, Amaratunga C, Beghain J, Langlois AC, et al (2014) A molecular marker of artemisinin-resistant *Plasmodium falciparum* malaria. Nature 505: 50–55. 10.1038/nature12876 24352242 PMC5007947

[3] Spangenberg T, Burrows JN, Kowalczyk P, McDonald S, Wells TN, et al (2013) The open access malaria box: a drug discovery catalyst for neglected diseases. PLoS One 8: e62906 10.1371/journal.pone.0062906 23798988 PMC3684613

[4] McGowan S (2013) Working in concert: the metalloaminopeptidases from *Plasmodium falciparum* . Curr Opin Struct Biol 23: 828–835. 10.1016/j.sbi.2013.07.015 23948130

[5] McGowan S, Oellig CA, Birru WA, Caradoc-Davies TT, Stack CM, et al (2010) Structure of the *Plasmodium falciparum* M17 aminopeptidase and significance for the design of drugs targeting the neutral exopeptidases. Proc Natl Acad Sci U S A 107: 2449–2454. 10.1073/pnas.0911813107 20133789 PMC2809755

[6] McGowan S, Porter CJ, Lowther J, Stack CM, Golding SJ, et al (2009) Structural basis for the inhibition of the essential *Plasmodium falciparum* M1 neutral aminopeptidase. Proc Natl Acad Sci U S A 106: 2537–2542. 10.1073/pnas.0807398106 19196988 PMC2636733

[7] Stack CM, Lowther J, Cunningham E, Donnelly S, Gardiner DL, et al (2007) Characterization of the *Plasmodium falciparum* M17 leucyl aminopeptidase. A protease involved in amino acid regulation with potential for antimalarial drug development. J Biol Chem 282: 2069–2080. 17107951 10.1074/jbc.M609251200

[8] Teuscher F, Lowther J, Skinner-Adams TS, Spielmann T, Dixon MW, et al (2007) The M18 aspartyl aminopeptidase of the human malaria parasite *Plasmodium falciparum* . J Biol Chem 282: 30817–30826. 17720817 10.1074/jbc.M704938200

[9] Harbut MB, Velmourougane G, Dalal S, Reiss G, Whisstock JC, et al (2011) Bestatin-based chemical biology strategy reveals distinct roles for malaria M1- and M17-family aminopeptidases. Proc Natl Acad Sci U S A 108: E526–534. 10.1073/pnas.1105601108 21844374 PMC3161592

[10] Ragheb D, Dalal S, Bompiani KM, Ray WK, Klemba M (2011) Distribution and biochemical properties of an M1-family aminopeptidase in *Plasmodium falciparum* indicate a role in vacuolar hemoglobin catabolism. J Biol Chem 286: 27255–27265. 10.1074/jbc.M111.225318 21659511 PMC3149319

[11] Deprez-Poulain R, Flipo M, Piveteau C, Leroux F, Dassonneville S, et al (2012) Structure-activity relationships and blood distribution of antiplasmodial aminopeptidase-1 inhibitors. J Med Chem 55: 10909–10917. 10.1021/jm301506h 23176597

[12] Kannan Sivaraman K, Paiardini A, Sienczyk M, Ruggeri C, Oellig CA, et al (2013) Synthesis and Structure-Activity Relationships of Phosphonic Arginine Mimetics as Inhibitors of the M1 and M17 Aminopeptidases from *Plasmodium falciparum* . J Med Chem 56: 5213–5217. 10.1021/jm4005972 23713488

[13] Skinner-Adams TS, Peatey CL, Anderson K, Trenholme KR, Krige D, et al (2012) The aminopeptidase inhibitor CHR-2863 is an orally bioavailable inhibitor of murine malaria. Antimicrob Agents Chemother 56: 3244–3249. 10.1128/AAC.06245-11 22450967 PMC3370795

[14] Velmourougane G, Harbut MB, Dalal S, McGowan S, Oellig CA, et al (2011) Synthesis of new (-)-bestatin-based inhibitor libraries reveals a novel binding mode in the S1 pocket of the essential malaria M1 metalloaminopeptidase. J Med Chem 54: 1655–1666. 10.1021/jm101227t 21366301 PMC3516848

[15] Sivaraman KK, Oellig CA, Huynh K, Atkinson SC, Poreba M, et al (2012) X-ray crystal structure and specificity of the *Plasmodium falciparum* malaria aminopeptidase *Pf*M18AAP. J Mol Biol 422: 495–507. 10.1016/j.jmb.2012.06.006 22709581

[16] Schüttelkopf AW, Aalten DMFv (2004) PRODRG—a tool for high-throughput crystallography of protein-ligand complexes. Acta Crystallogr D Biol Crystallogr D60. 10.1107/S0907444904011679 15272157

[17] Eswar N, Eramian D, Webb B, Shen M, Sali A (2008) Protein structure modeling with MODELLER. Methods Mol Biol 426: 145–159. 10.1007/978-1-60327-058-8_8 18542861

[18] Bramucci E, Paiardini A, Bossa F, Pascarella S (2012) PyMod: sequence similarity searches, multiple sequence-structure alignments, and homology modeling within PyMOL. BMC Bioinformatics Suppl 4:S2: Suppl 4:S2. 10.1186/1471-2105-13-S4-S2 22536966 PMC3303726

[19] Thomsen R, Christensen M (2006) MolDock: a new technique for high-accuracy molecular docking. J Med Chem 49: 3315–3321. 16722650 10.1021/jm051197e

[20] Mucha A, Drag M, Dalton JP, Kafarski P (2010) Metallo-aminopeptidase inhibitors. Biochimie 92: 1509–1529. 10.1016/j.biochi.2010.04.026 20457213 PMC7117057

[21] Rawlings ND, Barrett AJ, Bateman A (2012) MEROPS: the database of proteolytic enzymes, their substrates and inhibitors. Nucleic Acids Res 40: D343–350. 10.1093/nar/gkr987 22086950 PMC3245014

[22] Lipinski CA, Lombardo F, Dominy BW, Feeney PJ (1997) Experimental and computational approaches to estimate solubility and permeability in drug discovery and development settings. Adv Drug Del Rev 46: 3–26. 10.1016/s0169-409x(00)00129-0 11259830

[23] Agrawal A, Romero-Perez D, Jacobsen JA, Villarreal FJ, Cohen SM (2008) Zinc-binding groups modulate selective inhibition of MMPs. ChemMedChem 3: 812–820. 10.1002/cmdc.200700290 18181119 PMC2836234

