# Supplementary material for: Screening the Medicines for Malaria Venture "Malaria Box" against the Plasmodium falciparum Aminopeptidases, M1, M17 and M18
Source: PLoS One. 2015 Feb 20;10(2):e0115859. doi: 10.1371/journal.pone.0115859 (PMC4336144; doi:10.1371/journal.pone.0115859)
Supplement: S1 Fig — Enzyme activity that was reduced in comparison to control wells are indicated by “R” and compounds that had no effect on activity are shown as “UC” for unchanged. Compounds highlighted in grey are “drug-like” and the remainder are “probe-like”. (PDF) [file pone.0115859.s001.pdf]

COMPOUND	Set	Activity (PfA-M1 and/or PfA-M17)	Activity (PfM18A AP)	PLATE	WELL
MMV019066	D	UC	UC	MMV_PLATE_A	A02
MMV665941	P	UC	UC	MMV_PLATE_A	A03
MMV396680	P	UC	UC	MMV_PLATE_A	A04
MMV666601	P	UC	UC	MMV_PLATE_A	A05
MMV008294	P	UC	UC	MMV_PLATE_A	A06
MMV011259	D	UC	UC	MMV_PLATE_A	A07
MMV019406	P	UC	UC	MMV_PLATE_A	A08
MMV006278	D	UC	UC	MMV_PLATE_A	A09
MMV666688	P	UC	UC	MMV_PLATE_A	A10
MMV019110	D	UC	UC	MMV_PLATE_A	A11
MMV006427	D	UC	UC	MMV_PLATE_A	B02
MMV666062	P	UC	UC	MMV_PLATE_A	B03
MMV020885	P	UC	UC	MMV_PLATE_A	B04
MMV000570	P	UC	UC	MMV_PLATE_A	B05
MMV020439	D	UC	UC	MMV_PLATE_A	B06
MMV396672	D	UC	UC	MMV_PLATE_A	B07
MMV019871	D	UC	UC	MMV_PLATE_A	B08
MMV085583	P	UC	UC	MMV_PLATE_A	B09
MMV008416	P	UC	UC	MMV_PLATE_A	B10
MMV665874	D	UC	UC	MMV_PLATE_A	B11
MMV006203	P	UC	UC	MMV_PLATE_A	C02
MMV665977	P	UC	UC	MMV_PLATE_A	C03
MMV020549	D	UC	UC	MMV_PLATE_A	C04
MMV001246	D	UC	UC	MMV_PLATE_A	C05
MMV666607	P	UC	UC	MMV_PLATE_A	C06
MMV665915	D	UC	UC	MMV_PLATE_A	C07
MMV007695	P	UC	UC	MMV_PLATE_A	C08
MMV000448	P	UC	UC	MMV_PLATE_A	C09
MMV020500	D	UC	UC	MMV_PLATE_A	C10
MMV665878	D	UC	UC	MMV_PLATE_A	C11
MMV666101	P	UC	UC	MMV_PLATE_A	D02
MMV666596	P	UC	UC	MMV_PLATE_A	D03
MMV396679	P	UC	UC	MMV_PLATE_A	D04
MMV396797	D	UC	UC	MMV_PLATE_A	D05
MMV008138	D	UC	UC	MMV_PLATE_A	D06
MMV665916	D	UC	UC	MMV_PLATE_A	D07
MMV020788	P	UC	UC	MMV_PLATE_A	D08
MMV666691	P	R	UC	MMV_PLATE_A	D09
MMV665785	P	UC	UC	MMV_PLATE_A	D10
MMV665831	P	R	UC	MMV_PLATE_A	D11
MMV011099	D	UC	UC	MMV_PLATE_A	E02
MMV000642	P	UC	UC	MMV_PLATE_A	E03
MMV666600	P	UC	UC	MMV_PLATE_A	E04
MMV006172	P	UC	UC	MMV_PLATE_A	E05
MMV006309	P	R	UC	MMV_PLATE_A	E06
MMV006087	D	UC	UC	MMV_PLATE_A	E07
MMV020492	D	UC	UC	MMV_PLATE_A	E08
MMV006455	D	UC	UC	MMV_PLATE_A	E09
MMV665782	D	UC	UC	MMV_PLATE_A	E10
MMV665876	D	UC	UC	MMV_PLATE_A	E11
MMV666023	P	R	UC	MMV_PLATE_A	F02
MMV009063	D	UC	UC	MMV_PLATE_A	F03
MMV006558	P	UC	UC	MMV_PLATE_A	F04
MMV007160	P	UC	UC	MMV_PLATE_A	F05
MMV006429	D	UC	UC	MMV_PLATE_A	F06
MMV396703	D	UC	UC	MMV_PLATE_A	F07
MMV006937	D	UC	UC	MMV_PLATE_A	F08
MMV085203	P	UC	UC	MMV_PLATE_A	F09
MMV665820	D	UC	UC	MMV_PLATE_A	F10
MMV665841	P	UC	UC	MMV_PLATE_A	F11
MMV007116	D	UC	UC	MMV_PLATE_A	G02
MMV007384	P	UC	UC	MMV_PLATE_A	G03
MMV020548	D	UC	UC	MMV_PLATE_A	G04
MMV019258	D	UC	UC	MMV_PLATE_A	G05
MMV007686	P	UC	UC	MMV_PLATE_A	G06
MMV011256	D	UC	UC	MMV_PLATE_A	G07
MMV666693	D	UC	UC	MMV_PLATE_A	G08
MMV008956	D	UC	UC	MMV_PLATE_A	G09
MMV665827	P	UC	UC	MMV_PLATE_A	G10
MMV001038	D	UC	UC	MMV_PLATE_A	G11
MMV007839	D	R	UC	MMV_PLATE_A	H02
MMV000662	D	UC	UC	MMV_PLATE_A	H03
MMV396678	P	UC	UC	MMV_PLATE_A	H04
MMV006861	P	UC	UC	MMV_PLATE_A	H05
MMV006457	P	UC	UC	MMV_PLATE_A	H06
MMV396693	P	UC	UC	MMV_PLATE_A	H07
MMV011567	D	UC	UC	MMV_PLATE_A	H08
MMV007907	D	UC	UC	MMV_PLATE_A	H09
MMV665805	D	UC	UC	MMV_PLATE_A	H10
MMV666021	P	UC	UC	MMV_PLATE_A	H11
MMV665800	D	UC	UC	MMV_PLATE_B	A02
MMV000634	D	UC	UC	MMV_PLATE_B	A03
MMV666103	D	UC	UC	MMV_PLATE_B	A04
MMV666057	D	UC	UC	MMV_PLATE_B	A05
MMV007564	D	UC	UC	MMV_PLATE_B	A06
MMV001255	D	UC	UC	MMV_PLATE_B	A07
MMV665917	D	UC	UC	MMV_PLATE_B	A08
MMV000563	D	UC	UC	MMV_PLATE_B	A09
MMV665850	D	UC	UC	MMV_PLATE_B	A10
MMV665817	D	UC	UC	MMV_PLATE_B	A11
MMV665979	D	UC	UC	MMV_PLATE_B	B02
MMV665928	D	UC	UC	MMV_PLATE_B	B03
MMV666105	D	UC	UC	MMV_PLATE_B	B04
MMV666072	D	UC	UC	MMV_PLATE_B	B05
MMV000653	D	UC	UC	MMV_PLATE_B	B06
MMV000620	D	UC	UC	MMV_PLATE_B	B07
MMV665909	D	UC	UC	MMV_PLATE_B	B08
MMV665940	D	UC	UC	MMV_PLATE_B	B09
MMV665891	D	UC	UC	MMV_PLATE_B	B10
MMV665899	D	UC	UC	MMV_PLATE_B	B11
MMV665961	D	UC	UC	MMV_PLATE_B	C02
MMV665929	D	UC	UC	MMV_PLATE_B	C03
MMV666108	D	UC	UC	MMV_PLATE_B	C04
MMV665948	D	UC	UC	MMV_PLATE_B	C05
MMV006188	D	UC	UC	MMV_PLATE_B	C06
MMV001230	D	UC	UC	MMV_PLATE_B	C07
MMV665918	D	UC	UC	MMV_PLATE_B	C08
MMV665799	D	UC	UC	MMV_PLATE_B	C09
MMV665826	D	UC	UC	MMV_PLATE_B	C10
MMV665807	D	UC	UC	MMV_PLATE_B	C11
MMV665946	D	UC	UC	MMV_PLATE_B	D02
MMV665935	D	UC	UC	MMV_PLATE_B	D03
MMV666102	D	UC	UC	MMV_PLATE_B	D04
MMV666061	D	UC	UC	MMV_PLATE_B	D05
MMV008149	D	UC	UC	MMV_PLATE_B	D06
MMV019074	D	UC	UC	MMV_PLATE_B	D07
MMV665914	D	UC	UC	MMV_PLATE_B	D08
MMV665798	D	UC	UC	MMV_PLATE_B	D09
MMV665902	D	UC	UC	MMV_PLATE_B	D10
MMV665888	D	UC	UC	MMV_PLATE_B	D11
MMV666067	D	UC	UC	MMV_PLATE_B	E02
MMV665939	D	UC	UC	MMV_PLATE_B	E03
MMV009060	D	UC	UC	MMV_PLATE_B	E04
MMV666110	D	UC	UC	MMV_PLATE_B	E05
MMV019758	D	UC	UC	MMV_PLATE_B	E06
MMV000498	D	UC	UC	MMV_PLATE_B	E07
MMV665913	D	UC	UC	MMV_PLATE_B	E08
MMV665789	D	UC	UC	MMV_PLATE_B	E09
MMV665901	D	UC	UC	MMV_PLATE_B	E10
MMV666069	D	UC	UC	MMV_PLATE_B	E11
MMV666080	D	UC	UC	MMV_PLATE_B	F02
MMV666081	D	UC	UC	MMV_PLATE_B	F03
MMV666009	D	UC	UC	MMV_PLATE_B	F04
MMV019313	D	UC	UC	MMV_PLATE_B	F05
MMV019746	D	UC	UC	MMV_PLATE_B	F06
MMV019064	D	UC	UC	MMV_PLATE_B	F07
MMV011944	D	UC	UC	MMV_PLATE_B	F08
MMV665803	D	UC	UC	MMV_PLATE_B	F09
MMV665857	D	UC	UC	MMV_PLATE_B	F10
MMV666071	D	UC	UC	MMV_PLATE_B	F11
MMV019780	D	UC	UC	MMV_PLATE_B	G02
MMV666093	D	UC	UC	MMV_PLATE_B	G03
MMV665953	D	UC	UC	MMV_PLATE_B	G04
MMV000648	D	UC	UC	MMV_PLATE_B	G05
MMV019662	D	UC	UC	MMV_PLATE_B	G06
MMV007571	D	UC	UC	MMV_PLATE_B	G07
MMV007617	D	UC	UC	MMV_PLATE_B	G08
MMV665796	D	UC	UC	MMV_PLATE_B	G09
MMV665906	D	R	UC	MMV_PLATE_B	G10
MMV665954	D	UC	UC	MMV_PLATE_B	G11
MMV019738	D	UC	UC	MMV_PLATE_B	H02
MMV666075	D	UC	UC	MMV_PLATE_B	H03
MMV666070	D	UC	UC	MMV_PLATE_B	H04
MMV142383	D	UC	UC	MMV_PLATE_B	H05
MMV000788	D	UC	UC	MMV_PLATE_B	H06
MMV000561	D	UC	UC	MMV_PLATE_B	H07
MMV000248	D	UC	UC	MMV_PLATE_B	H08
MMV665879	D	UC	UC	MMV_PLATE_B	H09
MMV665890	D	UC	UC	MMV_PLATE_B	H10
MMV666116	D	UC	UC	MMV_PLATE_B	H11
MMV006913	D	UC	UC	MMV_PLATE_C	A02
MMV008127	D	UC	UC	MMV_PLATE_C	A03
MMV403679	D	R	UC	MMV_PLATE_C	A04
MMV305841	D	UC	UC	MMV_PLATE_C	A05
MMV019700	D	UC	UC	MMV_PLATE_C	A06
MMV019670	D	UC	UC	MMV_PLATE_C	A07
MMV001344	D	UC	UC	MMV_PLATE_C	A08
MMV011795	D	UC	UC	MMV_PLATE_C	A09
MMV019124	D	UC	UC	MMV_PLATE_C	A10
MMV006767	D	UC	UC	MMV_PLATE_C	A11
MMV007808	D	UC	UC	MMV_PLATE_C	B02
MMV019017	D	UC	UC	MMV_PLATE_C	B03
MMV396681	D	UC	UC	MMV_PLATE_C	B04
MMV006587	D	UC	UC	MMV_PLATE_C	B05
MMV019202	D	UC	UC	MMV_PLATE_C	B06
MMV000848	D	UC	UC	MMV_PLATE_C	B07
MMV020275	D	UC	UC	MMV_PLATE_C	B08
MMV019918	D	UC	UC	MMV_PLATE_C	B09
MMV075490	D	UC	UC	MMV_PLATE_C	B10
MMV396633	D	UC	UC	MMV_PLATE_C	B11
MMV007374	D	UC	UC	MMV_PLATE_C	C02
MMV396719	D	UC	UC	MMV_PLATE_C	C03
MMV396744	D	UC	UC	MMV_PLATE_C	C04
MMV006706	D	UC	UC	MMV_PLATE_C	C05
MMV009108	D	UC	UC	MMV_PLATE_C	C06
MMV020700	D	UC	UC	MMV_PLATE_C	C07
MMV007906	D	UC	UC	MMV_PLATE_C	C08
MMV008270	D	UC	UC	MMV_PLATE_C	C09
MMV019127	D	UC	UC	MMV_PLATE_C	C10
MMV396794	D	UC	UC	MMV_PLATE_C	C11
MMV396736	D	UC	UC	MMV_PLATE_C	D02
MMV306025	D	UC	UC	MMV_PLATE_C	D03
MMV056726	D	UC	UC	MMV_PLATE_C	D04
MMV274073	D	UC	UC	MMV_PLATE_C	D05
MMV018984	D	UC	UC	MMV_PLATE_C	D06
MMV000911	D	UC	UC	MMV_PLATE_C	D07
MMV007430	D	UC	UC	MMV_PLATE_C	D08
MMV007977	D	UC	UC	MMV_PLATE_C	D09
MMV020654	D	UC	UC	MMV_PLATE_C	D10
MMV665883	D	UC	UC	MMV_PLATE_C	D11
MMV084940	D	UC	UC	MMV_PLATE_C	E02
MMV396715	D	UC	UC	MMV_PLATE_C	E03
MMV000963	D	UC	UC	MMV_PLATE_C	E04
MMV006319	D	UC	UC	MMV_PLATE_C	E05
MMV000972	D	UC	UC	MMV_PLATE_C	E06
MMV020490	D	UC	UC	MMV_PLATE_C	E07
MMV001318	D	UC	UC	MMV_PLATE_C	E08
MMV007978	D	UC	UC	MMV_PLATE_C	E09
MMV020660	D	UC	UC	MMV_PLATE_C	E10
MMV665904	D	UC	UC	MMV_PLATE_C	E11
MMV396632	D	UC	UC	MMV_PLATE_C	F02
MMV007875	D	UC	UC	MMV_PLATE_C	F03
MMV006820	D	UC	UC	MMV_PLATE_C	F04
MMV396749	D	UC	UC	MMV_PLATE_C	F05
MMV011576	D	UC	UC	MMV_PLATE_C	F06
MMV020651	D	UC	UC	MMV_PLATE_C	F07
MMV000483	D	UC	UC	MMV_PLATE_C	F08
MMV019266	D	UC	UC	MMV_PLATE_C	F09
MMV001049	D	UC	UC	MMV_PLATE_C	F10
MMV665806	D	UC	UC	MMV_PLATE_C	F11
MMV667487	D	UC	UC	MMV_PLATE_C	G02
MMV000356	D	UC	UC	MMV_PLATE_C	G03
MMV396705	D	UC	UC	MMV_PLATE_C	G04
MMV006704	D	UC	UC	MMV_PLATE_C	G05
MMV000760	D	UC	UC	MMV_PLATE_C	G06
MMV007881	D	UC	UC	MMV_PLATE_C	G07
MMV008212	D	UC	UC	MMV_PLATE_C	G08
MMV007363	D	UC	UC	MMV_PLATE_C	G09
MMV007791	D	UC	UC	MMV_PLATE_C	G10
MMV665843	D	UC	UC	MMV_PLATE_C	G11
MMV396595	D	UC	UC	MMV_PLATE_C	H02
MMV396669	D	UC	UC	MMV_PLATE_C	H03
MMV396704	D	UC	UC	MMV_PLATE_C	H04
MMV019762	D	UC	UC	MMV_PLATE_C	H05
MMV020505	D	UC	UC	MMV_PLATE_C	H06
MMV020942	D	UC	UC	MMV_PLATE_C	H07
MMV000839	D	UC	UC	MMV_PLATE_C	H08
MMV666599	D	UC	UC	MMV_PLATE_C	H09
MMV000481	D	UC	UC	MMV_PLATE_C	H10
MMV665897	D	UC	UC	MMV_PLATE_C	H11
MMV665908	P	UC	UC	MMV_PLATE_D	A02
MMV665924	P	UC	UC	MMV_PLATE_D	A03
MMV665944	P	UC	UC	MMV_PLATE_D	A04
MMV666054	P	R	UC	MMV_PLATE_D	A05
MMV008474	P	UC	UC	MMV_PLATE_D	A06
MMV000445	P	UC	UC	MMV_PLATE_D	A07
MMV006882	P	UC	UC	MMV_PLATE_D	A08
MMV007127	P	UC	UC	MMV_PLATE_D	A09
MMV666123	P	UC	UC	MMV_PLATE_D	A10
MMV006389	P	UC	UC	MMV_PLATE_D	A11
MMV665949	P	UC	UC	MMV_PLATE_D	B02
MMV665934	P	UC	UC	MMV_PLATE_D	B03
MMV665994	P	UC	UC	MMV_PLATE_D	B04
MMV665980	P	UC	UC	MMV_PLATE_D	B05
MMV007577	P	UC	UC	MMV_PLATE_D	B06
MMV019995	P	UC	UC	MMV_PLATE_D	B07
MMV007208	P	UC	UC	MMV_PLATE_D	B08
MMV000442	P	UC	UC	MMV_PLATE_D	B09
MMV666124	P	UC	UC	MMV_PLATE_D	B10
MMV000444	P	UC	UC	MMV_PLATE_D	B11
MMV666109	P	UC	UC	MMV_PLATE_D	C02
MMV665936	P	UC	UC	MMV_PLATE_D	C03
MMV666020	P	UC	UC	MMV_PLATE_D	C04
MMV665971	P	UC	UC	MMV_PLATE_D	C05
MMV001241	P	UC	UC	MMV_PLATE_D	C06
MMV000720	P	R	UC	MMV_PLATE_D	C07
MMV000619	P	UC	UC	MMV_PLATE_D	C08
MMV006753	P	UC	UC	MMV_PLATE_D	C09
MMV006787	P	UC	UC	MMV_PLATE_D	C10
MMV665794	P	UC	UC	MMV_PLATE_D	C11
MMV000917	P	UC	UC	MMV_PLATE_D	D02
MMV666125	P	UC	UC	MMV_PLATE_D	D03
MMV665987	P	UC	UC	MMV_PLATE_D	D04
MMV007574	P	UC	UC	MMV_PLATE_D	D05
MMV000326	P	UC	UC	MMV_PLATE_D	D06
MMV000604	P	UC	UC	MMV_PLATE_D	D07
MMV007557	P	UC	UC	MMV_PLATE_D	D08
MMV000699	P	UC	UC	MMV_PLATE_D	D09
MMV009127	P	UC	UC	MMV_PLATE_D	D10
MMV665786	P	UC	UC	MMV_PLATE_D	D11
MMV006250	P	UC	UC	MMV_PLATE_D	E02
MMV666079	P	UC	UC	MMV_PLATE_D	E03
MMV665969	P	R	UC	MMV_PLATE_D	E04
MMV000304	P	UC	UC	MMV_PLATE_D	E05
MMV000443	P	UC	UC	MMV_PLATE_D	E06
MMV666604	P	UC	UC	MMV_PLATE_D	E07
MMV008455	P	UC	UC	MMV_PLATE_D	E08
MMV007199	P	UC	UC	MMV_PLATE_D	E09
MMV085471	P	UC	UC	MMV_PLATE_D	E10
MMV665797	P	UC	UC	MMV_PLATE_D	E11
MMV006513	P	UC	UC	MMV_PLATE_D	F02
MMV666095	P	UC	UC	MMV_PLATE_D	F03
MMV665943	P	UC	UC	MMV_PLATE_D	F04
MMV019555	P	UC	UC	MMV_PLATE_D	F05
MMV019741	P	UC	UC	MMV_PLATE_D	F06
MMV019690	P	UC	UC	MMV_PLATE_D	F07
MMV000621	P	UC	UC	MMV_PLATE_D	F08
MMV008173	P	UC	UC	MMV_PLATE_D	F09
MMV019241	P	R	UC	MMV_PLATE_D	F10
MMV665783	P	UC	UC	MMV_PLATE_D	F11
MMV000787	P	UC	UC	MMV_PLATE_D	G02
MMV666106	P	UC	UC	MMV_PLATE_D	G03
MMV666022	P	R	UC	MMV_PLATE_D	G04
MMV498479	P	UC	UC	MMV_PLATE_D	G05
MMV007396	P	UC	UC	MMV_PLATE_D	G06
MMV000617	P	UC	UC	MMV_PLATE_D	G07
MMV006764	P	UC	UC	MMV_PLATE_D	G08
MMV007275	P	UC	UC	MMV_PLATE_D	G09
MMV007273	P	R	UC	MMV_PLATE_D	G10
MMV666026	P	UC	UC	MMV_PLATE_D	G11
MMV665923	P	UC	UC	MMV_PLATE_D	H02
MMV666025	P	UC	UC	MMV_PLATE_D	H03
MMV666060	P	UC	UC	MMV_PLATE_D	H04
MMV666597	P	UC	UC	MMV_PLATE_D	H05
MMV007224	P	UC	UC	MMV_PLATE_D	H06
MMV020912	P	UC	UC	MMV_PLATE_D	H07
MMV007181	P	UC	UC	MMV_PLATE_D	H08
MMV007113	P	UC	UC	MMV_PLATE_D	H09
MMV007228	P	R	UC	MMV_PLATE_D	H10
MMV665972	P	UC	UC	MMV_PLATE_D	H11
MMV073843	P	UC	UC	MMV_PLATE_E	A02
MMV006303	P	UC	UC	MMV_PLATE_E	A03
MMV667490	P	UC	UC	MMV_PLATE_E	A04
MMV667492	P	UC	UC	MMV_PLATE_E	A05
MMV019881	P	UC	UC	MMV_PLATE_E	A06
MMV000478	P	UC	UC	MMV_PLATE_E	A07
MMV020403	P	UC	UC	MMV_PLATE_E	A08
MMV007764	P	UC	UC	MMV_PLATE_E	A09
MMV665830	P	UC	UC	MMV_PLATE_E	A10
MMV665886	P	UC	UC	MMV_PLATE_E	A11
MMV396664	P	UC	UC	MMV_PLATE_E	B02
MMV001239	P	UC	UC	MMV_PLATE_E	B03
MMV667491	P	UC	UC	MMV_PLATE_E	B04
MMV006169	P	UC	UC	MMV_PLATE_E	B05
MMV086103	P	UC	UC	MMV_PLATE_E	B06
MMV084434	P	R	UC	MMV_PLATE_E	B07
MMV666692	P	UC	UC	MMV_PLATE_E	B08
MMV665836	P	R	UC	MMV_PLATE_E	B09
MMV665881	P	UC	UC	MMV_PLATE_E	B10
MMV665875	P	UC	UC	MMV_PLATE_E	B11
MMV007020	P	UC	UC	MMV_PLATE_E	C02
MMV006656	P	UC	UC	MMV_PLATE_E	C03
MMV396726	P	UC	UC	MMV_PLATE_E	C04
MMV006522	P	UC	UC	MMV_PLATE_E	C05
MMV666686	P	UC	UC	MMV_PLATE_E	C06
MMV007474	P	UC	UC	MMV_PLATE_E	C07
MMV006962	P	UC	UC	MMV_PLATE_E	C08
MMV665813	P	UC	UC	MMV_PLATE_E	C09
MMV080034	P	UC	UC	MMV_PLATE_E	C10
MMV019199	P	UC	UC	MMV_PLATE_E	C11
MMV396652	P	UC	UC	MMV_PLATE_E	D02
MMV000704	P	UC	UC	MMV_PLATE_E	D03
MMV396635	P	UC	UC	MMV_PLATE_E	D04
MMV000986	P	UC	UC	MMV_PLATE_E	D05
MMV008160	P	UC	UC	MMV_PLATE_E	D06
MMV000753	P	R	UC	MMV_PLATE_E	D07
MMV011436	P	R	UC	MMV_PLATE_E	D08
MMV665882	P	UC	UC	MMV_PLATE_E	D09
MMV665809	P	UC	UC	MMV_PLATE_E	D10
MMV665810	P	UC	UC	MMV_PLATE_E	D11
MMV665927	P	UC	UC	MMV_PLATE_E	E02
MMV667486	P	UC	UC	MMV_PLATE_E	E03
MMV667488	P	UC	UC	MMV_PLATE_E	E04
MMV006825	P	UC	UC	MMV_PLATE_E	E05
MMV009085	P	UC	UC	MMV_PLATE_E	E06
MMV007591	P	UC	UC	MMV_PLATE_E	E07
MMV128432	P	UC	UC	MMV_PLATE_E	E08
MMV665852	P	UC	UC	MMV_PLATE_E	E09
MMV396717	P	R	UC	MMV_PLATE_E	E10
MMV638723	P	UC	UC	MMV_PLATE_E	E11
MMV396594	P	UC	UC	MMV_PLATE_E	F02
MMV396665	P	UC	UC	MMV_PLATE_E	F03
MMV396723	P	UC	UC	MMV_PLATE_E	F04
MMV007654	P	UC	UC	MMV_PLATE_E	F05
MMV011832	P	UC	UC	MMV_PLATE_E	F06
MMV020750	P	R	UC	MMV_PLATE_E	F07
MMV007092	P	UC	UC	MMV_PLATE_E	F08
MMV665898	P	UC	UC	MMV_PLATE_E	F09
MMV665864	P	UC	UC	MMV_PLATE_E	F10
MMV665814	P	R	UC	MMV_PLATE_E	F11
MMV007285	P	UC	UC	MMV_PLATE_E	G02
MMV396663	P	UC	UC	MMV_PLATE_E	G03
MMV007041	P	UC	UC	MMV_PLATE_E	G04
MMV000340	P	UC	UC	MMV_PLATE_E	G05
MMV666687	P	UC	UC	MMV_PLATE_E	G06
MMV645672	P	UC	UC	MMV_PLATE_E	G07
MMV011438	P	UC	UC	MMV_PLATE_E	G08
MMV665840	P	R	UC	MMV_PLATE_E	G09
MMV665894	P	UC	UC	MMV_PLATE_E	G10
MMV011522	P	UC	UC	MMV_PLATE_E	G11
MMV020243	P	UC	UC	MMV_PLATE_E	H02
MMV667489	P	UC	UC	MMV_PLATE_E	H03
MMV396770	P	R	UC	MMV_PLATE_E	H04
MMV009015	P	UC	UC	MMV_PLATE_E	H05
MMV008829	P	UC	UC	MMV_PLATE_E	H06
MMV011895	P	R	UC	MMV_PLATE_E	H07
MMV666689	P	UC	UC	MMV_PLATE_E	H08
MMV665824	P	UC	UC	MMV_PLATE_E	H09
MMV665812	P	UC	UC	MMV_PLATE_E	H10
MMV001041	P	R	UC	MMV_PLATE_E	H11

Figure S1: Primary screen of MMV400 using a multiplex aminopeptidase assay. Enzyme activity that was reduced in comparison to control wells are indicated by “R” and compounds that had no effect on activity are shown as “UC” for unchanged. Compounds highlighted in grey are “drug-like” and the remainder are “probe-like”.
