# Supplementary material for: Screening the Medicines for Malaria Venture "Malaria Box" against the Plasmodium falciparum Aminopeptidases, M1, M17 and M18
Source: PLoS One. 2015 Feb 20;10(2):e0115859. doi: 10.1371/journal.pone.0115859 (PMC4336144; doi:10.1371/journal.pone.0115859)
Supplement: S2 Fig — Enzyme activity in the presence of 100 μM compound (MMV# shown) was compared to activity of the enzyme in the absence of any inhibitor (-). An inhibitor control using Bestatin was included and no neutral aminopeptidase activity is detectable in the presence of 100 μM Bestatin. A dashed line is shown to indicate the when the activity of either enzyme was reduced by 90% or more. (PDF) [file pone.0115859.s002.pdf]

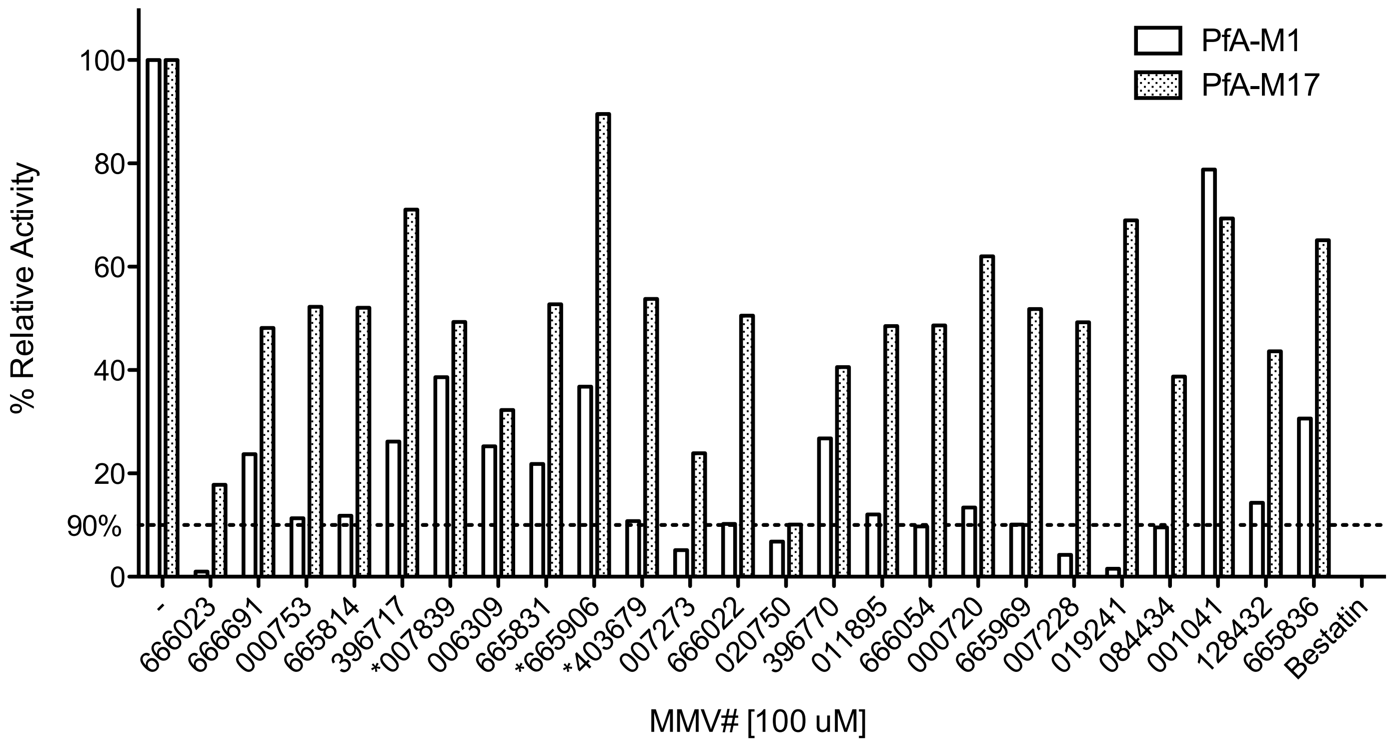

Figure S2: Secondary screen of *Pf*A-M1 and *Pf*A-M17 against 24 preliminary screen 'hits'. Enzyme activity in the presence of 100 μM compound (MMV# shown) was compared to activity of the enzyme in the absence of any inhibitor (-). An inhibitor control using Bestatin was included and no neutral aminopeptidase activity is detectable in the presence of 100 μM Bestatin. A dashed line is shown to indicate the when the activity of either enzyme was reduced by 90 % or more.
