# Supplementary material for: Screening the Medicines for Malaria Venture "Malaria Box" against the Plasmodium falciparum Aminopeptidases, M1, M17 and M18
Source: PLoS One. 2015 Feb 20;10(2):e0115859. doi: 10.1371/journal.pone.0115859 (PMC4336144; doi:10.1371/journal.pone.0115859)
Supplement: S3 Fig — (A) Enzyme activity in the presence of increasing inhibitor concentration. Numbers shown on curves are inhibitor concentration in μM. (B) Dixon plot for calculation of Ki where S1 and S2 are two different substrate concentrations (<< KM of enzyme). (PDF) [file pone.0115859.s003.pdf]

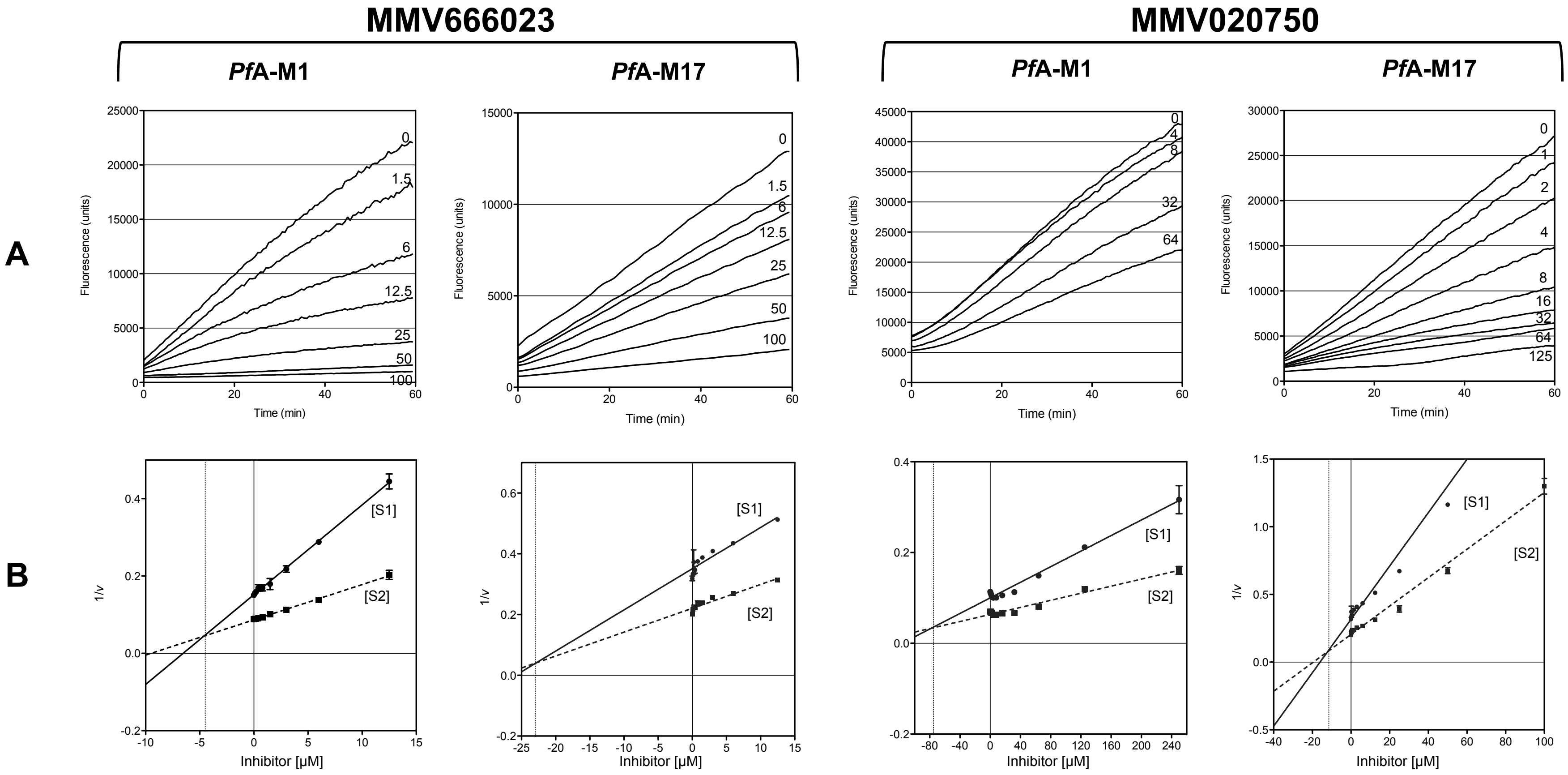

Figure S3: Inhibitory properties of MMV666023 and MMV020750. (A) Enzyme activity in the presence of increasing inhibitor concentration. Numbers shown on curves are inhibitor concentration in µM. (**B**) Dixon plot for calculation of K_i where [S1] and [S2] are two different substrate concentrations (<< K_M of enzyme).
