# Supplementary material for: Screening the Medicines for Malaria Venture "Malaria Box" against the Plasmodium falciparum Aminopeptidases, M1, M17 and M18
Source: PLoS One. 2015 Feb 20;10(2):e0115859. doi: 10.1371/journal.pone.0115859 (PMC4336144; doi:10.1371/journal.pone.0115859)
Supplement: S4 Fig — Dixon plots of Ki data shown in Fig. 5 (Ki defined as point of intersection and indicated by dotted line). Two different substrate concentrations are shown (solid circles and squares). Outliers not included in linear regression are shown as hollow squares or circles. 3D molecular docking diagrams shown with carbon atoms of PfA-M17 residues and the inhibitor are colored in light and dark gray, respectively. Zinc ions are shown as spheres. Corresponding 2D molecular docking representations shown on right hand panel. (PDF) [file pone.0115859.s004.pdf]

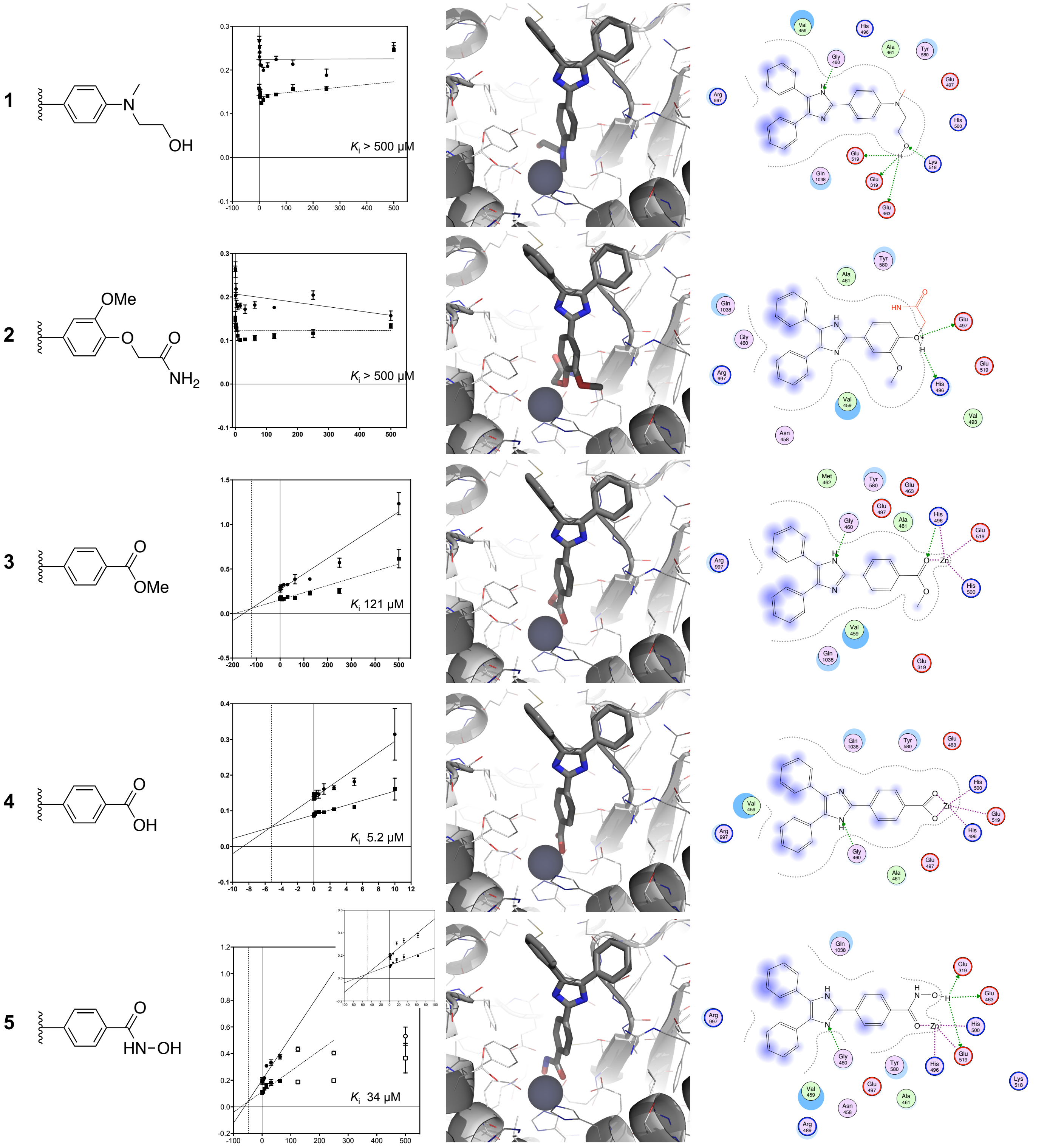

**Figure S4: MMV020750 derivatives binding to active sites of *Pf*A-M1.** Dixon plots of $K_i$ data shown in Table 2 ($K_i$ defined as point of intersection and indicated by dotted line). Two different substrate concentrations are shown (solid circles and squares). Outliers not included in linear regression are shown as hollow squares or circles. 3D molecular docking diagrams shown with carbon atoms of *Pf*A-M17 residues and the inhibitor are colored in light and dark gray, respectively. Zinc ions are shown as spheres. Corresponding 2D molecular docking representations shown on right hand panel.
