# Supplementary material for: Screening the Medicines for Malaria Venture "Malaria Box" against the Plasmodium falciparum Aminopeptidases, M1, M17 and M18
Source: PLoS One. 2015 Feb 20;10(2):e0115859. doi: 10.1371/journal.pone.0115859 (PMC4336144; doi:10.1371/journal.pone.0115859)
Supplement: S1 Methods — (PDF) [file pone.0115859.s006.pdf]

Methods S1

CHEMISTRY EXPERIMENTAL

General. Chemicals and solvents were purchased from standard suppliers and used without further purification. Davisil® silica gel (40-63µm), for flash column chromatography (FCC) was supplied by Grace Davison Discovery Sciences (Victoria, Australia) and deuterated solvents were purchased from Cambridge Isotope Laboratories, Inc. (USA, distributed by Novachem PTY. Ltd, Victoria, Australia).

Unless otherwise stated, reactions were carried out at ambient temperature. Reactions were monitored by thin layer chromatography on commercially available precoated aluminium-backed plates (Merck Kieselgel 60 F_{254}). Visualisation was by examination under UV light (254 and 366 nm). A solution of Fe(III)Cl_3 (5% in 0.5M $HCl_{(aq)}$) was used to visualise hydroxamic acids. All organic extracts collected after aqueous work-up procedures were dried over anhydrous Na_2SO_4 before gravity filtering and evaporation to dryness. Organic solvents were evaporated *in vacuo* at ≤ 40°C (water bath temperature).

^{1}H NMR and ^{13}C NMR spectra were recorded on a Bruker Avance Nanobay III 400MHz Ultrashield Plus spectrometer at 400.13 MHz and 100.62 MHz respectively. Chemical shifts (δ) are recorded in parts per million (ppm) with reference to the chemical shift of the deuterated solvent. Coupling constants (J) and carbon-fluorine coupling constants (J_{CF}) are recorded in Hz and the significant multiplicities described by singlet (s), doublet (d), triplet (t), quadruplet (q), broad (br), multiplet (m), doublet of doublets (dd), doublet of triplets (dt). Spectra were assigned using appropriate COSY, distortionless enhanced polarisation transfer (DEPT), HSQC and HMBC sequences.

LCMS were run to verify reaction outcome and purity using an Agilent 6120 Series Single Quad coupled to an Agilent 1260 Series HPLC. The following buffers were used; buffer A: 0.1% formic acid in H_2O; buffer B: 0.1% formic acid in MeCN. The following gradient was used with a Poroshell 120

EC-C18 50 x 3.0 mm 2.7 micron column, and a flow rate of 0.5 mL/min and total run time of 5 min; 0–1 min 95% buffer A and 5% buffer B, from 1-2.5 min up to 0% buffer A and 100% buffer B, held at this composition until 3.8 min, 3.8–4 min 95% buffer A and 5% buffer B, held until 5 min at this composition. Mass spectra were acquired in positive and negative ion mode with a scan range of 100–1000 *m/z*. UV detection was carried out at 214 and 254 nm. All retention times (t_R) are quoted in minutes.

All screening compounds were of > 95% purity unless specified in the individual monologue.

4-(4,5-Diphenyl-1*H*-imidazol-2-yl)-*N*-hydroxybenzamide (5). 4-(4,5-Diphenyl-1*H*-imidazol-2-yl)benzoic acid **(4)** (340 mg, 1.00 mmol) was dissolved in anhydrous tetrahydrofuran (5 mL), under an atmosphere of nitrogen at room temperature. To this stirred solution, was added carbonyldiimidazole (243 mg, 1.50 mmol, 1.5 eq), and the mixture stirred at room temperature for 2 hours. $NH_2OH.HCl$ (139 mg, 2.00 mmol, 2 eq) were then added, and the mixture allowed to stir at room temperature for 22 hours. LCMS analysis after this time indicated almost complete conversion had taken place. The mixture was diluted with saturated ammonium chloride solution (20 mL), before extraction with EtOAc (3 x 20 mL). The combined organic extracts were washed with brine (20 mL), and then dried over Na_2SO_4, before concentration under reduced pressure. The resulting crude residue was purified by flash column chromatography (eluent MeOH/DCM 0:100 to 10:90) to give several clean fractions. These were combined, and then concentrated under reduced pressure to give 157 mg of off-white solid. TLC analysis of the column fractions (MeOH/DCM 10:90) indicated a number of mixed fractions contained desired product were also present. These were combined and concentrated under reduced pressure. The resulting residue was then recrystallized from MeOH/water to give a further 100 mg of off-white solid. 1H-nmr and LCMS analysis indicated both solids were pure, and the desired target hydroxamic acid product. Total isolated yield: 257 mg (72%). ^1H NMR (400 MHz, DMSO) δ 12.83 (s, 1H), 11.27 (s, 1H), 9.07 (s, 1H), 8.15 (d, *J* = 8.4 Hz, 2H), 7.86 (d, *J* = 8.5 Hz, 2H), 7.62 – 7.49 (m, 4H), 7.46 (dd, *J* =

7.4/7.4 Hz, 2H), 7.39 (dd, J = 7.1/7.1 Hz, 1H), 7.31 (dd, J = 7.4/7.4 Hz, 2H), 7.23 (dd, J = 7.3/7.3 Hz, 1H); ^{13}C NMR (101 MHz, DMSO) δ 163.84, 144.66, 137.55, 134.97, 132.64, 132.05, 130.88, 128.86, 128.71, 128.48, 128.24, 127.96, 127.40, 127.10, 126.68, 124.90; *m/z* MS (TOF ES$^+$) $C_{22}H_{18}N_3O_2$ $[MH]^+$ calcd 356.1; found 356.2; MS (TOF ES$^-$) $C_{22}H_{16}N_3O_2$ $[M-H]^-$ calcd 354.1; found 354.1; LC-MS t_R: 3.32.
